# First observation of fluid-like eddy-dominant bursty bulk flow turbulence in the Earth’s tail plasma sheet

**DOI:** 10.1038/s41598-023-45867-w

**Published:** 2023-11-06

**Authors:** L. Q. Zhang, Chi. Wang, W. Baumjohann, R. S. Wang, J. Y. Wang, James L. Burch, Yu. V. Khotyaintsev

**Affiliations:** 1 State Key Laboratory of Space Weather, National Space Science Center, Chinese Academy of Sciences, Beijing, 100080 China; 2 Space Research Institute, Austrian Academy of Sciences, 8042 Graz, Austria; 3 CAS KCAS Key Laboratory of Geospace Environment, Department of Geophysics and Planetary Science, University of Science and Technology of China, Hefei, 230026 China; 4 Information Engineering College, Central University for Nationalities, Beijing, 100081 China; 5 Southwest Research Institute San Antonio, San Antonio, TX 78238 USA; 6 Swedish Institute of Space Physics, Uppsala, Sweden

**Keywords:** Space physics, Astronomy and planetary science

## Abstract

Turbulence is a ubiquitous phenomenon in neutral and conductive fluids. According to classical theory, turbulence is a rotating flow containing vortices of different scales. Eddies play a fundamental role in the nonlinear cascade of kinetic energy at different scales in turbulent flow. In conductive fluids, the Alfvénic/kinetic Alfvénic wave (AW/KAW) is the new “cell” of magnetohydrodynamic (MHD) turbulence (frozen-in condition). Wave energy, which has equal kinetic and magnetic energy, is redistributed among multiple-scale Fourier modes and transferred from the large MHD scale to the small kinetic scale through the collision of counter-propagating Alfvénic wave packages propagating along the magnetic field line. Fluid-like eddy-dominant plasma flow turbulence has never been found in space since the launch of the first satellite in 1957. In this paper, we report the first observation of eddy-dominant turbulence within magnetic reconnection-generated fast flow in the Earth’s tail plasma sheet by the Magnetospheric Multiscale Spacecraft (MMS). In eddy-dominant turbulent reconnection jet, ions dominate the flow field while electrons dominate current and magnetic fluctuations. Our findings shed new light on the nonlinear kinetic and magnetic energy cascade in MHD turbulence.

## Introduction

Magnetic reconnection occurs extensively in space, including on the surface of the Sun, in the magnetosheath region, and at the Earth’s dayside magnetopause and nightside magnetotail^1–3^. During the magnetic reconnection, the magnetic energy stored in the stretched thin current sheet is rapidly released and converted to kinetic energy. The fast flow generated during reconnection in the Earth’s magnetotail is also known as bursty bulk flow (BBF)^4–6^. BBF has been widely observed in the tail plasma sheet from the near-Earth region of ~ 7 R_E_ (R_E_ is the earth radius) to downtail beyond 100 R_E_^7,8^.

The BBF in the plasma sheet is a short-lived turbulent flow that lasts for several minutes to tens of minutes^9–11^. Typically, the BBF is convective in the central plasma sheet (CPS) when β > 1 (where β is the ratio of thermal pressure to magnetic pressure), and field-aligned near the plasma sheet boundary layer (PSBL) when 0.1 < β < 1^12,13^. In the vicinity of the reconnection region, the speed of the BBF can reach up to ~ 1000 km/s. However, after leaving the reconnection source region, the BBF may experience significant deceleration, slowing down to several hundred kilometers per second before reaching the braking region around ~ 10 R_E_^14,15^.

The BBF is intrinsically a turbulent plasma flow with superimposed eddies and waves. Magnetic fluctuations during the BBF demonstrate enhanced wave activities within the turbulence^16–19^. Simultaneous measurements of electric and magnetic fluctuations show an increase in the parallel-predominantly Poynting flux (**P** = **∆E × ∆B**) and power spectral ratio |**∆**E|/|**∆**B during the BBF interval. These features are consistent with those of the kinetic Alfvénic wave (KAW)^20,21^. Additionally, compressible fluctuations induced by slow-mode waves are also found within the turbulent BBF^22,23^.

Early research on velocity fluctuations in the tail plasma sheet revealed the formation of coherent vorticity (**ω** = **∇ × V**, where V is the velocity of the mass center of the fluid element) and large-scale vortex structures^24–28^. Recent direct measurements of plasma vorticity based on four-point joint observations by MMS spacecraft have exhibited an enhancement in ω within the turbulent BB^29,30^. The ω-field in the course of the BBF exhibits perpendicular-anisotropy. In particular, low-frequency fluctuations in the ω-field show a good correlation with the flow speed of the BBF. The higher the flow velocity, the greater the vorticity. The properties of vorticity, namely convective or kinetic, depends on the flow filed of the BBF. Kinetic BBF tends to have stronger vorticity than convective BBF^31^.

In this paper, we analyze the velocity and magnetic fluctuations observed within the BBF observed on Aug 16, 2018, by the MMS spacecraft. MMS mission^32^ consists of four identical spacecraft, with high-accuracy measurements of plasma moments and small spacecraft separations of tens of kilometers. The MMS data has 0.125-s resolution for Fluxgate magnetometers (FGM)^33^, 4.5-s resolution for Fast Plasma Investigation (FPI)^34^, and 0.03-s resolution for Electric Field Double Probe (EDP)^35^. Geocentric Solar Magnetospheric (GSM) coordinate system is adopted. During this event, the BBF near the PSBL lasted for 22 min and had an average flow velocity of 312 km/s (V_A_ = 937 km/s, and ${{V}_{A}=\pm\, {\mathrm{B}}_{0}\left({\mu }_{0}\rho \right)}^{-1/2}$ is Alfvénic velocity), making it a sub-Alfvénic flow. The velocity fluctuations exhibited parallel-anisotropy (∆V_//_/∆V_⊥_ ~ 2.1) and had low correlation with the magnetic fluctuations. The ω-field in the course of the BBF is perpendicular-predominantly. In particular, the spectra of magnetic field B and current J demonstrate dissipation scaling in the inertial range (below ion gyrofrequency). These observations offer a new perspective on the kinetic energy cascade and dissipation by eddies in magnetohydrodynamic (MHD) turbulence.

### A scenario of eddy and wave contributions to the velocity fluctuations within BBF turbulence

**Figure 1:**
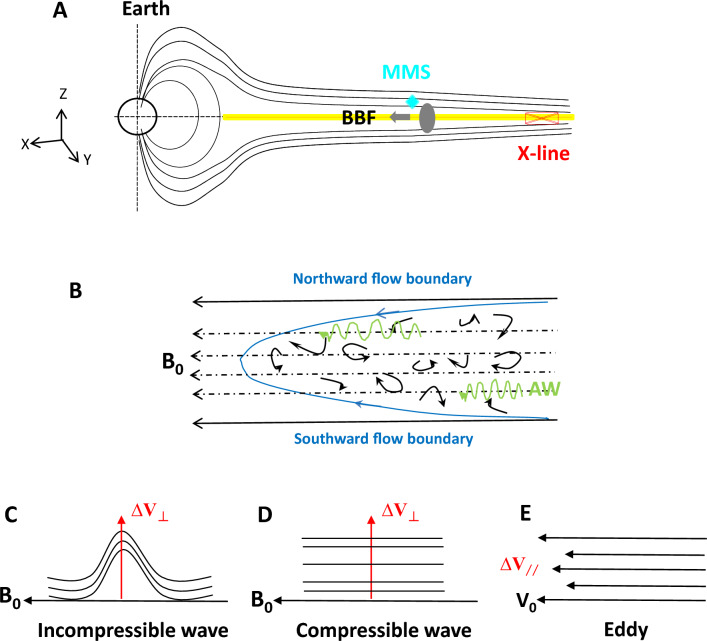
Schematic diagram of the field-aligned BBF turbulence superimposing eddy and wave at the PSBL away from the reconnection source region. (**A**) Is a schematic of the earthward-moving BBF along the background magnetic field at the inner PSBL (Geocentric solar magnetospheric (GSM) coordinates are adopted). The yellow region marks the cross-tail current sheet at the center of the tail plasma sheet, and the X-line denotes the location of the reconnection source region. MMS is located in the northward hemisphere and encounters the turbulent BBF near the PSBL. (**B**) Depicts the scenario of eddies of different scales within the BBFs. The black solid straight line with an arrow represents the magnetic field lines at the flow boundaries in the north and south hemispheres, and the dashed straight line with an arrow represents the magnetic field line internal to the BBF. Blue represent flow boundary. Green represents Alfvénic waves along the background magnetic field. Near the flow boundary, the flow and background field are both earthward, and the eddies are frozen in the flow, moving with the BBF together. The AW travels through the BBF along the B_0_. (**C**) Shows the perpendicular velocity and magnetic fluctuations caused by the Alfvénic mode. (**D**) Shows the perpendicular velocity fluctuation and parallel magnetic fluctuations caused by compressible modes. (**E**) Depicts the parallel velocity fluctuation (∆V_//_) caused by eddies.

The scenario of velocity fluctuations in the BBF turbulence at the boundary layer of the plasma sheet is illustrated in Fig. 1. After leaving the reconnection source region, the BBF moves earthward. In the center of the BBF, the current sheet typically has a significant normal field ((B_z_) and/or dawn-dusk field (B_y_), resulting in mainly convective motion in the CPS. However, at the plasma sheet boundary layer, the Bx component becomes dominant, causing the BBF to become field-aligned and move along the background magnetic field lines **B**_**0**_ (Panel A).

Eddies and waves coexist in the BBF (Panel B). Eddies of various scales are frozen in the BBF and move together (based on the Taylor hypothesis). These eddies contribute to the velocity fluctuations in the flow field of the BBF. Alfvén waves travel through the BBF at an Alfvénic speed along the B_0_ and contribute to magnetic and velocity fluctuations, both in the perpendicular direction relative to B_0_ (Panel C). Compressible modes may also exist, causing perpendicular fluctuations in the flow field and parallel fluctuations in the magnetic field. The rotating eddies cause enhanced ω within the BBF, primarily in the perpendicular direction relative to the background flow or, equivalently, the magnetic field (Panel E). Considering that only eddy contributes to parallel fluctuation ∆V_//_, there has,

1 $$ \Delta {\mathbf{V}}_{//} = {\mathbf{V}}_{{//{\text{eddy}}}} , $$

2 $$ \Delta {\mathbf{V}}_{ \bot } = \Delta {\mathbf{V}}_{{ \bot {\text{eddy}}}} + \Delta {\mathbf{V}}_{{ \bot {\text{wave}}}} . $$

The Alfvénic perturbations satisfy the relationship: $\Delta \mathbf{E}+ \Delta {\mathbf{V}}_{\perp \mathrm{wave}}\times {{\varvec{B}}}_{0}=0$ and $\frac{\Delta {\varvec{E}}}{\Delta {\mathbf{B}}_{\perp }}{=V}_{A}$. Here $\Delta \mathbf{E}$ is the perturbed electric field. Simple calculation yields that: $\Delta {\mathbf{V}}_{\perp \mathrm{wave}}=\frac{\Delta {\mathbf{B}}_{\perp }}{{B}_{0}}{V}_{A}$. Inserting it into Eq. (2), we obtain

3 $$\frac{\Delta {\mathbf{V}}_{\perp }}{{V}_{A}}=\frac{\Delta {\mathbf{V}}_{\perp \mathbf{e}\mathbf{d}\mathbf{d}\mathbf{y}}}{{V}_{A}}+\frac{\Delta {\mathbf{B}}_{\perp }}{{B}_{0}}.$$

The first and second terms at the left hand represent the contributions of eddy and wave to ∆V_⊥_, respectively.

Defining parallel Alfvén Mach number ${\mathrm{M}}_{\mathrm{A}//}=\frac{|\updelta {\mathbf{V}}_{//}|}{{\mathbf{V}}_{\mathrm{A}}}$ and perpendicular Alfvén Mach number ${\mathrm{M}}_{\mathrm{A}\perp }=\frac{|\updelta {\mathbf{V}}_{\perp }|}{{\mathbf{V}}_{\mathrm{A}}}$, the MHD turbulence is eddy-dominant if M_A//_ > M_A⊥_ and/or M_A⊥_ > $\frac{\Delta {\mathbf{B}}_{\perp }}{{B}_{0}}$. Else, the MHD turbulence is wave-dominant.

## Event on Aug 16, 2018

### Overview

**Figure 2:**
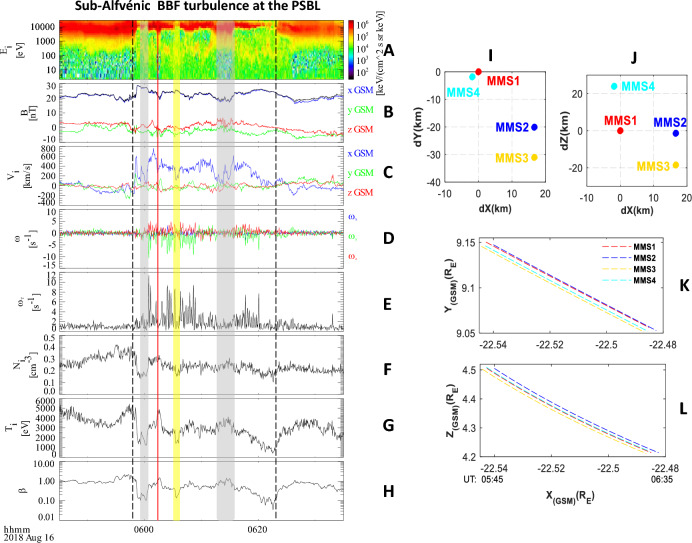
Overview on sub-Alfvénic BBF turbulence at the boundary layer of the plasma sheet observed by MMS1 spacecraft. The left column shows the temporal evolution of the turbulent BBF. (**A**) Ion energy spectrum. (**B**) Bx, By, and Bz. (**C**) Vx, Vy, and Vz. (**D**) ω_x,_ ω_y_ and ω_z_. (**E**) Total ω ω_T_. (**F**) Ion density n_i_. (**G**) Ion temperature T_i_. (**H**) Plasma β. The right column is the orbit of the MMS. Panels (**I,J**) show the distances between MMS1 and the other three MMS satellites in the X–Y and X–Z planes, respectively. Panels (**K,L**) show the trajectories of MMS from 05:30 to 06:30 UT in the X–Y and X–Z planes, respectively. The BBF lasts from 05:58 to 06: 21 UT (between the two vertical lines). Shadows mark the opposite flow of the low-ω superimposed on the normal flow of the BBF. The grey shadows are the opposite flows of higher-β, and yellow shadow is the opposite flows of lower-β. The red vertical line marks the short dip of MMS1 into the CPS at 06:04 UT.

Figure 2 shows the temporal evolution of ion and magnetic field measurements obtained by the FPI and FGM instruments onboard the MMS1 spacecraft from 05:45 to 06:35 UT. The trajectory and separation distance of the MMS spacecraft are also shown. The MMS spacecraft is initially located in the dusk-side plasma sheet around (− 22.5 R_E_, 9.1 R_E_, 4.5 R_E_). The separation distance between the MMS1 and MMS2 (MMS3) spacecraft is 17 km in the X–Y plane, while the separation distance between MMS1 and MMS4 is 2 km in the X–Y plane. In the X–Z plane, the separation distance between MMS1 and MMS2 (MMS3) is 20 km, while the separation distance between MMS1 and MMS1 is 31 km in the same plane.

The BBF begins at 05:58 UT and ends at 06:21 UT, as indicated by the two vertical lines in the figure. During this time, the MMS spacecraft moves slowly radially towards Earth (Panel K) and towards the equator in the north–south direction (Panel L). Prior to the appearance of the BBF, the plasma sheet is slightly flapping, and the MMS1 spacecraft gradually moves towards the CPS. Upon entering the BBF, the spacecraft moves towards the inner PSBL, where the ion density is approximately ~ 0.3 cm^−3^ and the ion temperature is approximately 3 keV. At 06:04 UT, MMS1 briefly enters the CPS and then returns to the inner plasma sheet.

The BBF is a sub-Alfvénic flow with an average velocity of 312 km/s (V_A_ = 937 km/s). The flow field in the course of the BBF is highly structured, with opposing flows against the BBF on a temporal scale of 1–3 min (marked by shadows). In the normal flow field of the BBF (outside the shadows), the velocity fluctuates strongly, and the ω field shows significant enhancement. In contrast, the opposing flow with large negative V-fluctuation (ΔV < 0) has only slight velocity fluctuations and no ω-enhancement. The ion population (Panel A) exhibits subtle variations in the opposing flows of higher-β and lower-β. The lower-β opposing flow (yellow shadow) has stronger B and lower n/T and corresponds to a decrease in ion flux at high-energy (above 10 keV) in the ion energy spectrum. The higher-β opposing flow (grey shadows) has weaker B and higher n/T and corresponds to an increase in ion flux in low and medium energy (500 eV–5 keV). The opposing flow of mixed different ion populations is consistent with the passage of large-scale Kelvin–Helmholtz vortex structures^36,37^.

### Parallel-anisotropic velocity fluctuation

**Figure 3:**
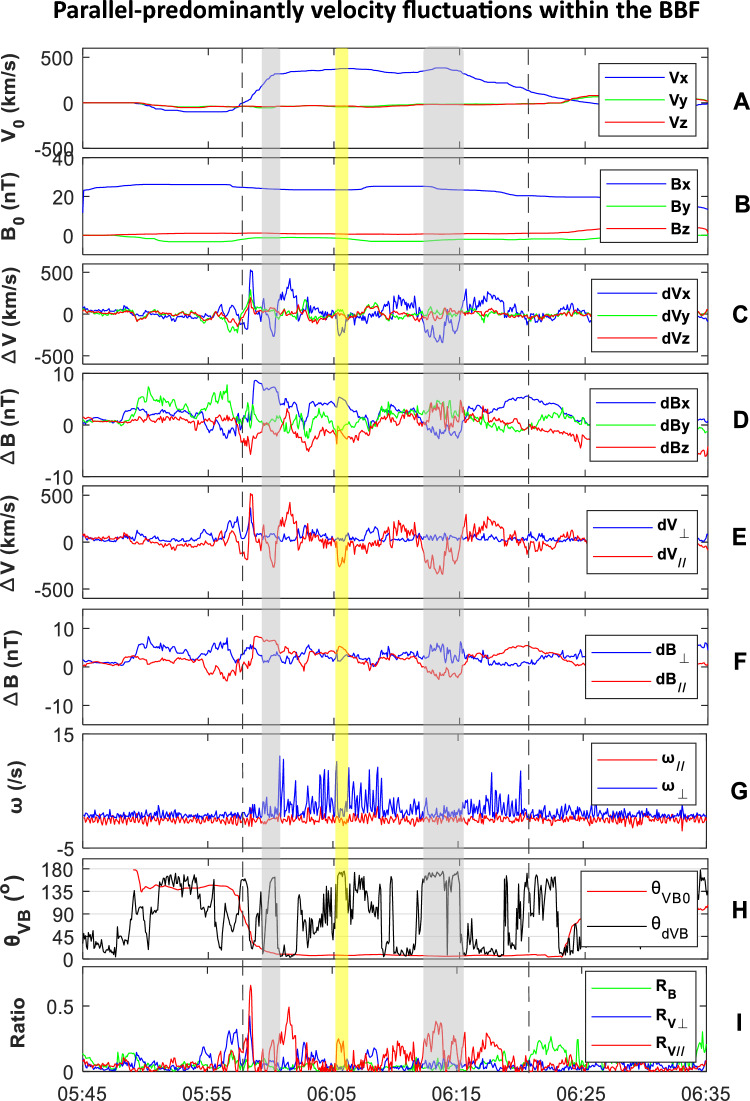
Perturbed and unperturbed fluctuations in the flow and magnetic fields during the interval of the parallel BBF near the PSBL observed by MMS1. (**A**) V_x0_, V_y0_, and V_z0_. (**B**) B_x0_, B_y0_, and B_z0_. (**C**) ∆V_x_, ∆V_y_, and ∆V_z_. (**D**) ∆B_x_, ∆B_y_, and ∆B_z_. (**E**) ∆V_//_ and ∆V_⊥_. (**F**) ∆B_//_ and ∆B_⊥_. (**G**) ω_//_, and ω_⊥_. (**H**) θ_VB0_ (= arctan(V_0_/B_0_)) and θ_dVB_ (= arctan(∆V/B_0_)) (**I**) R_B_ (= ∆B_⊥_/B_0_), Rv_//_ (= ∆V_//_/V_A_) and Rv_⊥_(= ∆V_⊥_/V_A_). The median filter with a cutoff frequency of 0.015 Hz is applied to separate the unperturbed and perturbed components in the velocity and magnetic fields. The high-resolution magnetic field data (B-data) is interpolated to match the FPI-data. Subsequently, the interpolated magnetic field data is used to calculate the perturbed and unperturbed V_//_/ω_//_ and V_⊥_/ω_⊥_ components. The shadows are the same as in Fig. 2.

Unperturbed and perturbed fluctuations in the flow and magnetic fields during the BBF interval are shown in Fig. 3. The BBF turbulence exhibits a quiet background. From Panel B, it can be seen that the background magnetic field is dominated by the B_x0_ component, which slowly decreases from 28.4 to 22.3 nT, with an average value of 23.7 nT. In comparison, the B_y0_ and B_z0_ components are small and unclear, indicating that the background plasma sheet is in a quiet state. This rules out the potential influence of plasma sheet motion on the BBF turbulence, such as tilting, twisting, and flapping. The V_x0_ component remains steady at 304 km/s (V_A_ = 937 km/s), with small fluctuations in V_y0_ and V_z0_. The average angle between V_0_ and B_0_ is approximately 2.7°, indicating that the background flow of the BBF is quasi-parallel, moving along the background magnetic field.

Panel E shows that the velocity fluctuation in the turbulent flow field exhibits a prominent parallel-anisotropy (∆V_//_/∆V_⊥_ ~ 2.1), with an average value of ∆V/V_0_ over the BBF time of ~ 0.5. The Mach number (M_A_ = V/C, where V is flow velocity and C is speed of sound)) is ~ 0.15. The magnetic fluctuation in the BBF is slight (∆B/B_0_ ~ 0.16), without clear preference in parallel and perpendicular directions (∆B_//_/∆B_⊥_ ~ 1.0). The ω-field of the eddy-dominant BBF is perpendicular-predominantly.

To clarify the contributions of eddies and waves to BBF turbulence, we further compare the different terms in Formula (3). Panel (I) displays M_A//_, M_A⊥_, and R_B_ (= ∆B_⊥_/B_0_). Throughout the BBF interval, R_B_ remains small. M_A//_ is mostly much greater than M_A⊥_, and both are much greater than R_B_. Clearly, eddy dominates the velocity fluctuation in the flow field of the BBF, as opposed to wave. At the end of the BBF, R_B_ becomes the greatest, indicating that wave fluctuations dominate the post-BBF flow.

### E-fluctuations and wave activities

**Figure 4:**
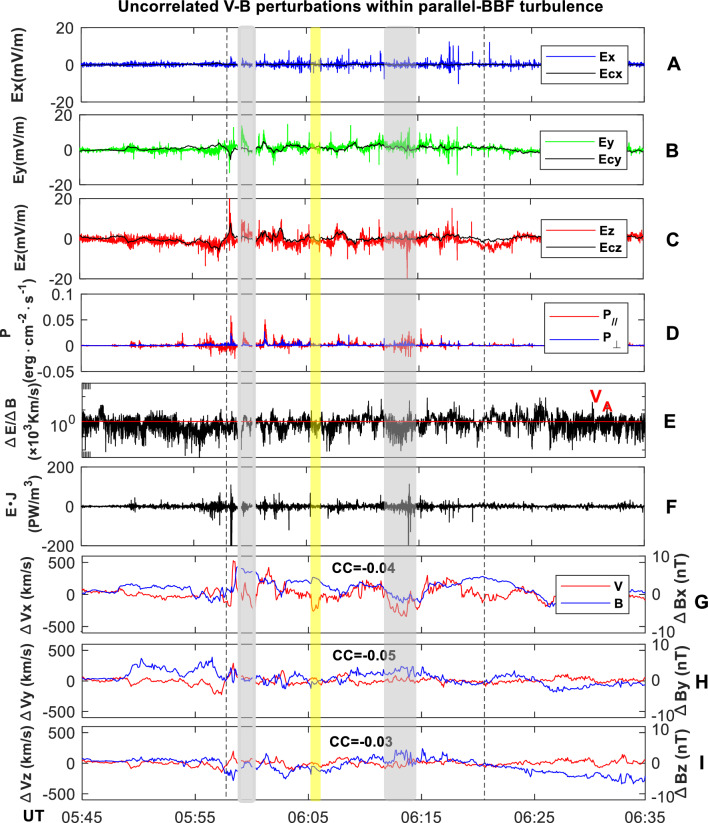
E-fluctuation and wave actives within eddy-dominant BBF turbulence. (**A**) Measured Ex vs. Ecx. (**B**) Measured Ey vs. Ecy. (**C**) Measured Ez vs. Ecz. (**D**) P_//_ and P_⊥_. (**E**) |∆E_⊥_/∆B_⊥_|. (**F**) **E J**. ∆B_z_. (**G**) ∆V_x_ vs. ∆B_x_. (**H**) ∆V_y_ vs. ∆B_y_. (**I**) ∆V_z_ vs. ∆B_z_. To obtain the convective and kinetic **E** (**E**_**k**_ = **E** + **E**_**c**_, and **Ec** = **V × B**), the original **E** and **B** data are all interpolated too match the 4.5 s time cadence of the FPI data. The interpolated **E**/**B** data is then used to calculate the **Ec** and **E**_**k**_. To calculate the Poynting flux, the original **E**-data is interpolated to match the B-data. The electric and magnetic fields data from the EDP and FGM instruments onboard MMS1 are detrended by a 10-min-window running average. The perturbed **E** and **B** fields are then used to calculate the Poynting vector ($\mathbf{P}=\Delta \mathbf{E}\times \Delta {\varvec{B}}$). To accurately calculate the correlation coefficient (CC) in Panels (**F–H**), only data during the BBF-time are used. A total of 304 data points are collected (from 05:58 to 06:21).

The associated evolutions of **E**-field and Poynting flux (**P**) from 05:40 to 06:30 UT are plotted in Fig. 4. All components in the E-field fiercely fluctuate, with spikes of 10–20 mV/m. Despite the spikes, the E-field is composed of a superimposition of slowly-varying convective-E (**E**_**c**_) and rapidly-varying kinetic-**E** (**E**_**k**_), with **E**_**c**_ being the main component. The net E⋅J over the BBF time is negative (− 0.9), suggesting a Joule dissipation of the BBF.

The Poynting flux shows a substantial enhancement within the BBF, with the enhanced-P having a higher parallel component (**P**_**//**_) than the perpendicular component (**P**_**⊥**_). The maximum magnitude of P_//_ is ~ 0.06 erg cm^−2^ s^−1^. Notably, the strengths of P in the normal flow (outside shadows) and opposite flow (inside shadows) show no distinct difference. This implies that the AW/KAW activities during the course of the BBF may not be directly related to the ω-field.

The correlation coefficient (CC) between velocity and magnetic fluctuations is shown in Panels F–H. During the BBF interval, the CC is 0.04 between ∆V_x_ and ∆B_x_, 0.05 between ∆V_y_ and ∆B_y,_ and 0.03 between ∆V_z_ and ∆B_z_. The CC between ∆V and ∆B is evaluated to be ~ 0.2. The low correlation indicates that within the eddy-dominant BBF, magnetic and velocity fluctuations are non-Alfvénic in nature.

### Non-Kolmogorov spectrum and electron dynamic

**Figure 5:**
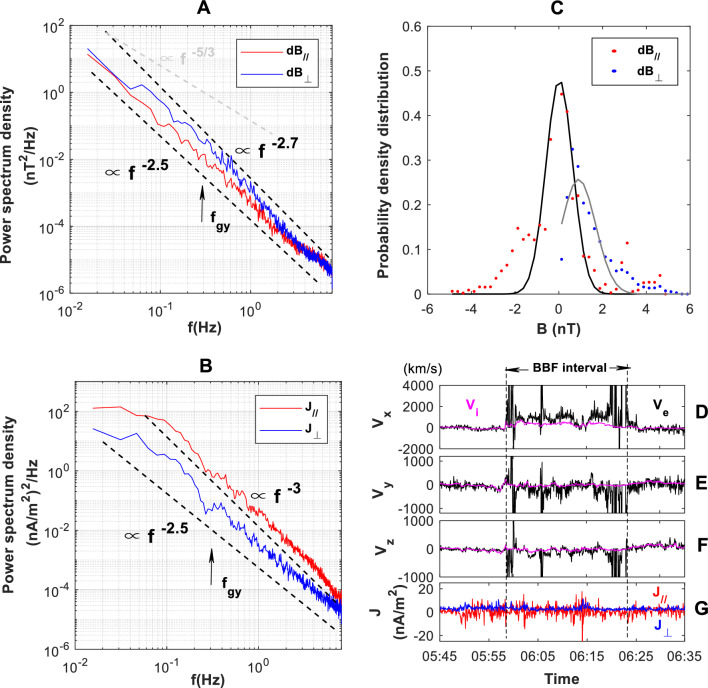
Spectrum and electron dynamic in the eddy-dominant BBF turbulence. Ion gyro-frequency f_gy_ is 0.3 Hz. (**A**) Spectra of ∆B_//_ and ∆B_⊥_. (**B**) Spectra of J_//_ and J_⊥_ (calculated by **∇ × B**/*μ*_0_). (**C**) Power density probability of the ∆B_//_ and ∆B_⊥_. The lines are fitted with a single Gaussian (black and grey) that provides an estimate of intermittency in BBF turbulence. (**D**) v_ix_, v_ex_. (**E**) v_iy_, v_ey_. (**F**) v_iz_,v_ez_. (**G**) J_//_ and J_⊥_ (calculated by **∇ × B**/*μ*_0_).

Figure 5A illustrates the power spectrum of the ∆B_//_ and ∆B_⊥_. Below 0.3 Hz (ion gyrofrequency, f_gy_), the B-spectrum deviates significantly from Kolmogorov’s − 5/3 law. The scaling of the spectra of ∆B_//_ and ∆B_⊥_ is similar, approximately − 2.5. Above 0.3 Hz, the scaling of the ∆B_//_-spectrum remains the same, whereas the scaling of the ∆B_⊥_-spectrum becomes − 2.7, indicating faster dissipation of magnetic energy in the perpendicular direction than in the parallel direction.

The spectra of J_//_ and J_⊥_ are shown in Panel (B). Similarly, the J-spectrum deviates from Kolmogorov’s − 5/3 law in the inertial regime. At 0.28 Hz, a deep dip appears in the ∆J_⊥_-spectrum. The dip in the ∆J_//_-spectrum is ambiguous. In the low-frequency subrange, the J_⊥_-spectrum rises from ~ 10 (nA/m^2^)^2^/Hz at 0.03 Hz to ~ 20 (nA/m^2^)^2^/Hz at 0.05 Hz. Above 0.05 Hz, the J_⊥_-spectrum is straight with a slope of about − 2.5. Below 0.05 Hz, the J_//_-spectrum is flat. From 0.05 Hz to 0.1 Hz, the spectrum of the J_//_ slowly decrease from ~ 70 (nA/m^2^)^2^/Hz to ~ 50 (nA/m^2^)^2^/Hz. Above 0.1 z, the J_//_-spectrum has a dissipation scaling of − 3-like.

Panel (C) shows probability density distributions (PDD) of ∆B_//_ and ∆B_⊥_. The distribution of the ∆B_⊥_ deviates from the Gaussian distribution with a clear tendency. When |∆B_⊥_| < 1. 5 nT, the |∆B_⊥_| is lower than the Gaussian fitting result, while when |∆B_⊥_| > 1. 5 nT, it is higher than the result of Gaussian fitting result. In contrast, the distribution of ∆B_//_ is basically symmetric. When |∆B_//_| < 1. 5 nT, ∆B_//_ basically follows Gaussian distribution. However, when |∆B_//_| > 1. 5 nT, it is distinctly higher than the expect value of Gaussian fitting. For both ∆B_//_ and ∆B_⊥_, the greater fluctuation has a higher probability, which is consistent with the intermittence characteristic. Therefore, the intermittent turbulence is a likely explanation for the non-Kolmogorov spectra of ∆B_//_ and ∆B_⊥_ in the inertial subrange^38–40^.

The temporal evolution of electron velocity (measured by FPI) and momentum current from 05:58 to 06:21 UT are shown in Panels (D-G). Momentum current** J** is calculated by **J** = n_i_e (**V**_**i**_ − **V**_**e**_), where the subscript i and e represents ion and electron, respectively, n_i_ is the ion density, and e is the ion charge. For all components, **V**_**e**_ fluctuated fiercely, and it is about an order of magnitude higher than V_i_. Thus, ions and electrons are decoupled in their macroscopic fluid behavior. Electron dominates the turbulent current **J**. Panel G shows that the electron-dominated **J** has a greater parallel component (J_//_) than perpendicular component (J_⊥_) which accounts for the J_//_ being higher than J_⊥_ in Panel B.

### Eddy-dominant BBF on July 30, 2017

**Figure 6:**
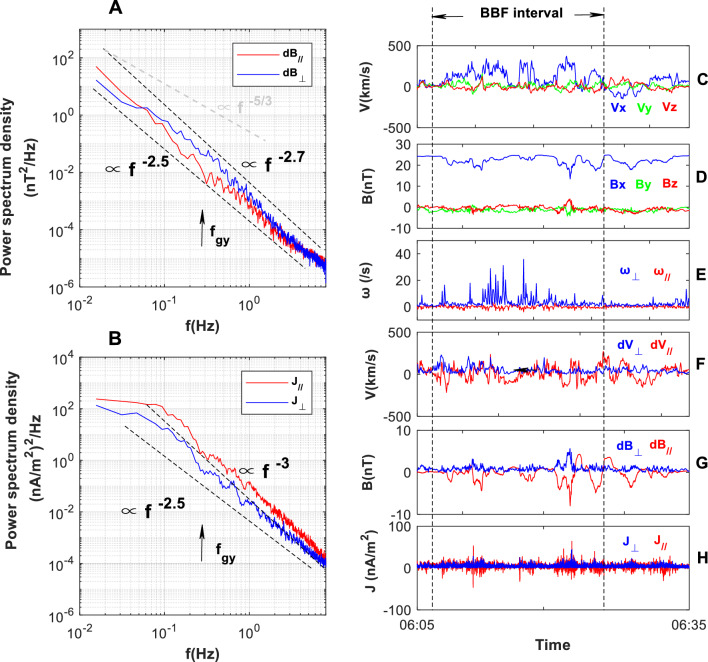
Spectral of B and J in the eddy-dominant BBF turbulence at the PSBL on July 30, 2017 observed by MMS1. The BBF lasts from 06:09 to 06:28 (within the two vertical lines). Left column is power spectra. (**A**) Spectra of ∆B_//_ and ∆B_⊥_. (**B**) Spectra of J_//_ and J_⊥_. Right column is the evolution of the flow and magnetic field in the course of the BBF. (**C**) Vx, Vy, and Vz (from ion measurement by FPI). (**D**) Bx, By, and Bz. (**E**) ω_//_, and ω_⊥_. (**F**) ∆V_//_ and ∆V_⊥_. (**G**) ∆B_//_ and ∆B_⊥_. (**H**) J_//_ and J_⊥_ (**J** = **∇ × B**/*μ*_0_).

To verify the non-Kolmogorov spectrum, we present another eddy-dominant BBF observed at the PSBL on July 30, 2017. The two BBFs have similar properties in their flow and magnetic fields. The temporal evolutions of the flow and magnetic fields from 06:05 to 06:35 UT are shown in Fig. 6 (right column). MMS1 is initially posited in the midnight near-Earth plasma sheet at (− 16.5 R_E_, 2.0 R_E_, 4.7 R_E_). The BBF appears at 06:09 UT and lasts until 06:28 UT. The average velocity of the BBF is ~ 155 km/s, and the maximum velocity is 423 km/s (V_A_ = 1066 km/s). Therefore, the BBF is a sub-Alfvénic flow. The V_x_ component dominated the flow field, and the B_x_ component dominated the magnetic field. The B_y_ and B_z_ components in the background plasma sheet were quite weak. The flow field is highly turbulent (∆V/V_0_ ~ 0.8). The velocity fluctuation in the turbulent flow field is parallel-anisotropic (∆V_//_/∆V_⊥_ ~ 1.9), with a low correlation with the magnetic fluctuation (CC = 0.06). Thus, the BBF is dominated by the eddy. The magnetic turbulent fluctuation was parallel-anisotropic (∆B_//_/∆B_⊥_ ~ 1.9). Additionally, the eddy-dominant BBF has a stronger J_//_ component than the J_⊥_ component (calculated by **∇ × B**/*μ*_0_, where μ_0_ is the magnetic permeability of the plasma).

The power spectra of B and J are shown in Panels A and B. In the low-frequency subrange, both parallel and perpendicular spectra of **B** as well as **J** deviate from Kolmogorov’s − 5/3 law, in a similar dissipative slope. In the high-frequency subrange, the slope of the ∆B_⊥_-spectrum is steeper than the ∆B_//_-spectrum, while the slope of the J_//_-spectrum is steeper than the J_⊥_-spectrum.

Comparing Figs. 5 and 6, the main spectra characteristics of two BBFs are similar, including their spectral intensities and slopes. This similarity suggests that the non-Kolmogorov spectra in the inertial range are not accidental. In the eddy-dominant BBF, both eddy and wave can subtract energy from magnetic field. As a result, magnetic energy dissipation may be faster than predicted by Kolmogorov’s − 5/3 law. This may be responsible for the dissipative scaling of the **B** and **J** spectra in the inertial range.

### Eddy-dominant MHD turbulence: physics and implication

Near the PSBL, the magnetic field is strong, and the plasma tends to move along the magnetic field. In this case, the fluid-like flow turbulence may occur, in which the flow fluctuates mainly along the background flow as if the magnetic field does not exist.

The Alfvénic speed near the PSBL is typically of ~ 1000 km/s. While propagating through the BBF of several hundred kilometers per second, the difference in velocities of wave and flow results in a large “Doppler shift”, causing the wave to be non-resonant with the turbulent eddies. This non-resonance prevents the wave from interacting with the turbulence, as the turbulence is not able to effectively “catch up” with the wave. As a consequence, the eddy overwhelms the wave and dominates the flow within the BBF.

Unlike Alfvénic vorticity in the wave turbulence^41–44^, the eddy vorticity in the BBF turbulence is perpendicular-predominantly. The presence of non-Alfvénic vorticity in the BBF implies that there are independent kinetic and magnetic cascades occurring in MHD turbulence^45–47^. That is to say, the kinetic and magnetic cascades are not strongly coupled and can evolve independently of each other within BBF turbulence.

## Summary

In summary, eddies dominate over wave within the parallel BBF at the PSBL, resulting in velocity fluctuations that are primarily parallel and have low correlation with magnetic fluctuations. The ω-field in the eddy-dominant BBF is predominantly perpendicular. While ions dominate the flow field, electrons dominate current and magnetic fluctuations. Specifically, at low frequencies, the spectra of both magnetic field and current exhibit non-Kolmogorov scaling in the inertial range. At high frequencies (above the ion gyrofrequency), the B_⊥_-spectrum has a steeper slope than the B_//_-spectrum, while the J_//_-spectrum has a steeper slope than the J_⊥_-spectrum.

## Data Availability

The datasets analyzed during the current study are available in the [CDAWEB] repository, [https://cdaweb.gsfc.nasa.gov/pub/data/mms/].
